# uORFs: Important *Cis*-Regulatory Elements in Plants

**DOI:** 10.3390/ijms21176238

**Published:** 2020-08-28

**Authors:** Ting Zhang, Anqi Wu, Yaping Yue, Yu Zhao

**Affiliations:** National Key Laboratory of Crop Genetic Improvement, Huazhong Agricultural University, Wuhan 430070, China; zhangtingxianyun@webmail.hzau.edu.cn (T.Z.); wuanqi527@webmail.hzau.edu.cn (A.W.); yueyaping@webmail.hzau.edu.cn (Y.Y.)

**Keywords:** uORFs, translational regulation, metabolic pathways, morphogenesis, disease resistance, nutrient absorption, plant breeding

## Abstract

Gene expression is regulated at many levels, including mRNA transcription, translation, and post-translational modification. Compared with transcriptional regulation, mRNA translational control is a more critical step in gene expression and allows for more rapid changes of encoded protein concentrations in cells. Translation is highly regulated by complex interactions between *cis*-acting elements and *trans*-acting factors. Initiation is not only the first phase of translation, but also the core of translational regulation, because it limits the rate of protein synthesis. As potent *cis*-regulatory elements in eukaryotic mRNAs, upstream open reading frames (uORFs) generally inhibit the translation initiation of downstream major ORFs (mORFs) through ribosome stalling. During the past few years, with the development of RNA-seq and ribosome profiling, functional uORFs have been identified and characterized in many organisms. Here, we review uORF identification, uORF classification, and uORF-mediated translation initiation. More importantly, we summarize the translational regulation of uORFs in plant metabolic pathways, morphogenesis, disease resistance, and nutrient absorption, which open up an avenue for precisely modulating the plant growth and development, as well as environmental adaption. Additionally, we also discuss prospective applications of uORFs in plant breeding.

## 1. Introduction

Plant growth and development is a precise and highly regulated process, which is controlled at multiple steps, including mRNA transcription, splicing, stability, and translation. In recent years, with the advances in microarray, qRT-PCR, and RNA-seq, great progress has been made in understanding transcriptional regulation, and many transcriptional regulatory networks centered on transcription factors have been constructed. However, changes in some gene transcriptional levels do not correlate with protein stability or function [1,2]. Changes in the protein content or activity can directly affect proteomics, enabling organisms to make reversible and rapid responses to cellular signals or environmental stimuli. Therefore, it is essential to unravel the regulatory mechanisms related to protein content, activity, or mRNA translation.

The translation of eukaryotic mRNA is a complicated multi-step process that is mainly composed of initiation, elongation, and termination phases. Among them, translation initiation is the primary rate-limiting phase and involves a remarkably extended set of eukaryotic initiation factors (eIFs) providing for the complex regulation of gene expression. The canonical translation of eukaryotic mRNAs is initiated via the m^7^G cap-dependent scanning mechanism [3,4,5], that is, the translation machines scan from the 5′-cap of the mRNA base-by-base to search for the AUG initiation codon. Therefore, whether the translation machines can move smoothly or scan the AUG initiation codon of the major reading open frame (mORF) of mRNA largely depends on the composition and structure of the 5′-UTR (untranslated region) of mRNA [6,7].

uORFs (upstream open reading frames), as translational control elements, lie in the 5′-UTR of eukaryotic mRNAs. uORFs can repress the translation initiation of mORFs, which are downstream of the uORFs [8], and regulate the translation initiation rates of mORFs by isolating ribosomes or affecting the mRNA stability through nonsense-mediated decay (NMD) [8]. Many previous studies show that uORFs are prevalent in eukaryotic mRNAs [9,10,11,12,13,14]. For example, approximately 50% of human and mouse protein-encoding genes contain one or more uORFs, and the presence of uORFs correlates with reduced protein levels [15,16,17,18,19,20]. While, in plants, uORFs have been found in 24–30% of the 5′-UTR region of mRNAs [21,22], regulatory functions of only a few uORFs have been identified and characterized; however, their regulatory mechanisms are poorly understood. Therefore, it is essential to further explore uORFs in plants and analyze their translational regulatory mechanisms, which could help deepen our understanding of plant developments and adaptations to changing environments. In this review, we summarize the identification and classification of uORFs and the regulatory mechanism of uORFs in plant developmental processes and responses to various stresses. This will further enrich our knowledge of the regulatory mechanisms behind plant developments and the interactions of plants and environments and help us diversify plant-breeding methods by the directional transformation of plants at the translation level.

## 2. Identification of uORFs

uORFs are a kind of small ORFs whose start codons are located upstream of mORF start codons. The canonical AUG start codon sequence functions as the translation initiation site of most uORFs, and AUG uORFs typically act as repressors. Recently, genome-wide ribosome-profiling studies suggest that thousands of uORFs also initiate with non-AUG start codons, such as CUG, UUG, and GUG, most of which have smaller functions on reporter gene expressions [23,24,25]. Ribosome profiling, a high-throughput technique, has been widely used for the genome-wide identification of uORFs [26,27,28]. In addition, bioinformatics can also be useful for the retrieval of uORF information. For example, uORF-Finder, a Perl program developed by Hayden et al. [11], has been applied to identify conserved uORFs in *Arabidopsis thaliana* and rice transcripts. uORFSCAN, a comparative R-nomics program, has also been used to find conserved uORFs in rice, wheat, sorghum, and *Arabidopsis* [12]. An online tool called uORFlight (http://uorflight.whu.edu.cn/home.html) [29] and a web-based database called PsuORF (http://psorf.whu.edu.cn/) [30] can also be available for searching out uORFs in plants. During recent years, it has been predicted and reported that there are 10,104 uORFs in *Arabidopsis* [31], 21,915 uORFs in maize [32], 1329 uORFs in tomatoes [33], 10,226 in yeast [34], and 35,735 uORFs in *Drosophila melanogaster* [35] and that uORF-containing mRNAs in human, mouse, and zebrafish, respectively, account for about 49%, 44%, and 50% of their total mRNAs [36,37,38]. Collectively, these findings suggest that uORFs are widespread *cis*-elements in eukaryotic protein-coding genes (Table 1), and these uORF identifications will further advance our understanding of translational control mechanisms in eukaryotes.

## 3. Classification of uORFs

**Figure 1:**
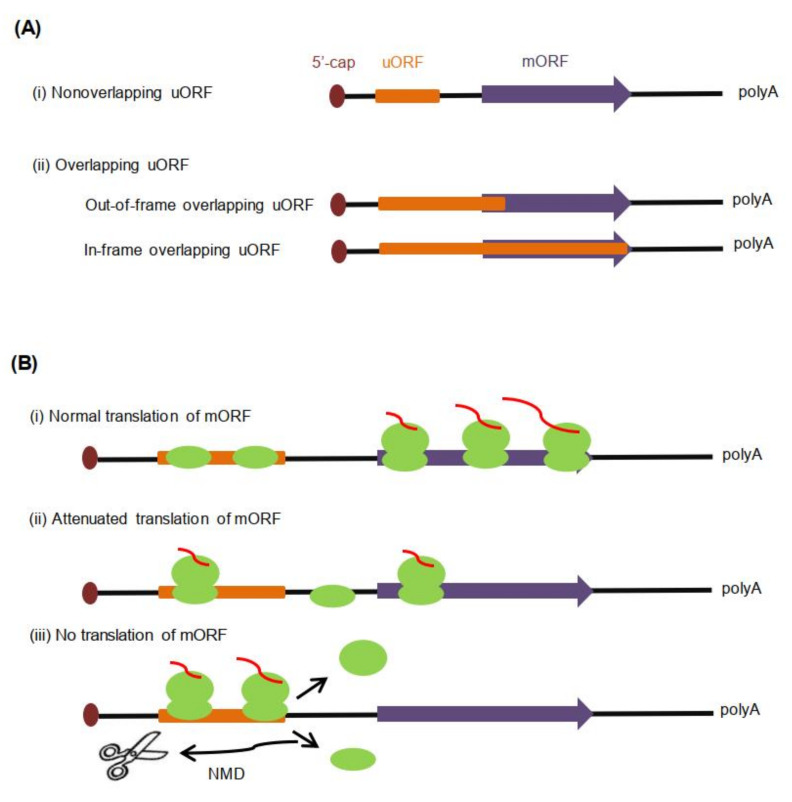
Upstream open reading frame (uORF) classifications in eukaryotes (including plants) and mechanisms of their influence on main ORF (mORF) translation. (**A**). Classification of uORFs based on the relative positions of the uORF (orange) and the mORF (purple). (**B**). Regulatory mechanisms of uORF to mORF translation. (i) When uORFs are not recognized by ribosomes, mORF translation is normal. (ii) When uORFs are translated by ribosomes, and then at the translation termination phase, 40S small subunits remain bound to the mRNA to reinitiate mORF translation. mORF translation is generally significantly lower, because uORF translation usually interferes with other scanning ribosomes [54]. (iii) If both small and large ribosomes dissociate from mRNA or trigger NMD (nonsense-mediated mRNA decay) by ribosome stalling at the end of uORF translation, mORF will not be translated.

Based on the relative positions of uORF termination codons and mORF initiation codon, uORFs in eukaryotes can be divided into two categories (Figure 1A): (i) Nonoverlapping uORFs; about 85% of identified uORFs fall into this category [39]. Stop codons of this kind of uORFs are located upstream of mORF AUG start codon (mAUG), and the length of the interval sequences between the stop codons of uORFs and mAUG is variable. Most of the current studies focus on this kind of uORF. (ii) Overlapping uORFs, the stop codons of which are downstream of mAUG. Overlapping uORFs can be further divided into two subgroups. One is out-of-frame overlapping uORFs, the reading frames of which are inconsistent with those of the mORF [8]. If uORFs are recognized and translated by scanning ribosomes, the mORF translation will not occur due to the difference of the reading frames [40]. Therefore, out-of-frame overlapping uORFs have a significant inhibitory function on mORF expression. The other is in-frame overlapping uORFs, which have reading frames consistent with the mORF and share the same stop codon with the mORF [8]. Only when the translated uORFs do not overlap with the mORF, can the mORF be recognized and translated by the translation initiation complex [41].

According to whether the regulatory function of uORFs depends on their sequences, these uORFs can be divided into two categories [42]: (i) Sequence-independent uORFs. The function of these uORFs is only affected by the uORF location or sequences around the uAUG (start codon of a uORF) [11]. Most of identified uORFs so far belong to this category. Among them, minimum ORFs are very special and referred to as “AUG-stops”, because AUG start codon is directly followed by a stop codon [43]. (ii) Sequence-dependent uORFs. The uORFs contain rare codons that make ribosomes move more slowly [44,45] or make metabolites act as effector molecules to cause ribosome stalling at the stop codons of the uORFs [46,47]. In order to further identify these nascent regulatory peptides and to understand the prevalence of these regulatory uORFs in eukaryotes, uORFs that encoded similar amino acid sequences are named as “conserved peptide uORFs (CPuORFs)”, and they have been explored among the homologous genes of different organisms or paralogous genes of the same species. On the basis of the conservation pattern of amino acid sequences, plant CPuORFs are further grouped into two classes: Class I CPuORFs—C-terminal amino acid sequences within them are evolutionarily conserved, while Class II CPuORFs amino acid sequences are globally conserved or the N-terminal and/or middle regions are conserved. A study suggested that the interaction between a regulatory peptide and the exit tunnel components might exist when ribosome stalling occurred at the uORF stop codon [48]. Therefore, it is speculated that Class I CPuORFs might encode regulatory peptides.

## 4. uORF-Mediated Regulation of Translation Initiation

Eukaryotic translation initiation mainly depends on the m^7^G cap-dependent manner [49,50]. First, a ternary complex that is composed of eukaryotic initiation factor 2 (eIF2), GTP, and initial tRNA (Met-tRNA_i_^Met^) is formed and binds to the 40S ribosomal subunit to assemble the 43S preinitiation complex (PIC). eIF4E, the m^7^G cap-binding protein, can bind to the 5′-cap of the mRNA and recruit eIF4G and eIF4A. The ATP-dependent helicase activity of eIF4A is activated by eIF4G, so that eIF4A can unwind the secondary structures of the 5′-UTR of mRNA. Then, the 43S PIC binds to the 5′-end of the assembled mRNA and scans along the 5′-end of the mRNA to search for the AUG start codon. When the anticodon of the Met-tRNA_i_^Met^ successfully recognizes the AUG start codon of the mRNA, various translation initiation factors are released accompanying the hydrolysis of GTP. Finally, the 60S ribosomal subunit combines to form the complete 80S translation initiation complex.

The composition and structure of the mRNA 5′-UTR usually affects the efficiency of the mRNA translation [6,7]. As for uORF-containing mRNA, there are three alternative fates of mORF: normal translation, reduced translation, or no corresponding translation product [8] (Figure 1B). Leaky scanning of uORFs [51,52,53] can ensure the proper translation of the mORF; once the uORFs are recognized and translated by PIC, mORF translation may be reduced or blocked, which is related to the state of ribosomes in mRNA. If the 40S small subunits remain bound to the mRNA after termination of the uORF translation, they may be assembled into full ribosomes again, then continue to scan to reinitiate mORF translation. However, the translation level of mORFs with the uORFs is generally significantly lower than those without uORFs, because the translation of uORFs usually interferes with other scanning ribosomes [54]. Conversely, downstream mORFs will not be translated because of NMD triggered by ribosome stalling or the failure of the reinitiation translation caused by ribosome dissociation from the mRNA. Generally, functional uORFs can affect the running state of ribosomes in mRNA and mORF translation.

The premise of uORF translations is that uAUGs can be recognized by ribosomes and that the AUG start codon with a high translation efficiency is usually in a favorable Kozak sequence (Given that the first nucleotide of AUG start codon is +1, the Kozak sequence usually flanks the AUG start codon from −6 to +4 and can be summarized as RXXAUGG; R can be A or G). Compared with mAUG, uAUGs are generally surrounded by the unfavorable Kozak sequence contexts, and more preferable sequence contexts around uAUGs result in the higher translational efficiency of uORFs [8,55]. Studies indicate that the length of uORFs is not a major factor that influences uORF functions [43], but the further uAUGs are from the 5′-cap or the closer the stop codons of uORFs are from the mAUG, the more repressive uORFs are [8,55]. Furthermore, ribosome profiling during the life cycle of *D. melanogaster* demonstrated that the translational efficiency of genes with a single translated uORF only declined by 8.38–30.4%, whereas genes with multiple translated uORFs declined by 18.4–60.7%, which suggests that uORFs cumulatively inhibited the translations of genes [35]. For sequence-dependent uORFs, if the amino acid sequences of uORFs are changed, their ability of translation inhibition maybe decreases or disappears [56,57,58]. Thus, the inhibitory function of uORFs may be influenced by the sequence context around uAUGs, their amino acid sequences and location, and the number of uORFs. The effects of the first two factors have also been well-characterized in plants so far.

## 5. uORFs as Translation Regulators in Plants

### 5.1. uORF-Mediated Translational Regulation of Plant Metabolic Pathways

In plants, small metabolites are often used as signal molecules and play a variety of roles, so it is essential to maintain their contents and relative stabilities.

Polyamines (PAs), ubiquitous, positively charged metabolites, include putrescine (Put), spermidine (Spd), and spermine (Spm) [59], which participate in N:C balance maintenance, growth and physiology, and stress responses in plants [60,61]. The aminopropyl group, produced by S-adenosylmethionine decarboxylase (AdoMetDC; EC 4.1.1.50) [62,63], is an important component whose continuous supply ensures PA biosynthesis. In *Arabidopsis*, *AdoMetDC* mRNA 5′-UTR contains two highly conserved overlapping “tiny” uORF and “small” uORF, which are translated in a PA-dependent manner. The length of the tiny uORF is 12 bp, while the small uORF is 156 bp. It is noteworthy that there is only one nucleotide overlap between the two uORFs. When the PA level is low, the short peptide translated by the tiny uORF blocks the translation of small uORF, which consequently ensures the translation of mORF, thus favoring the synthesis of PAs. Under higher PA conditions, the translation machine either leaks the weaker uAUG (belonging to a tiny uORF) and then recognizes the stronger uAUG (belonging to a small uORF), or the translation machine recognizes the weaker uAUG but then, at once, undergoes −1 frameshifting for translating the repressive small uORF, followed by the translational repression of mORF, which decrease the PA synthesis [64]. In other words, the normal translation of PA-responsive gene *AdoMetDC* depends on the tiny uORF, while constitutively repressing the expression of *AdoMetDC* at the translation level without increased PA levels relies on a small peptide encoded by a small uORF.

It is well-known that catabolism of PAs is mainly the oxidative deamination of Spd and Spm, which is catalyzed by flavin-containing polyamine oxidases (PAOs, EC 1.5.3.3) in plants. In the *Arabidopsis thaliana* genome, five PAO-encoding genes have been characterized, which exhibit differences in their 5′-UTRs [60,65]. *AtPAO2* and *AtPAO3* transcripts have only one uORF in their 5′-UTR regions [11], and *AtPAO4* contains two uORFs [66]. Functional analyses indicate that the *AtPAO2* uORF is vital for the translational control of the *AtPAO2* mORF. The *GUS* reporter gene driven by the native promoter fused to the uORF-containing UTR of *AtPAO2* (*AtPAO2prom-uORF::GUS*) showed prominent translational repression compared with that by the native promoter fused to UTR without the uORF (*AtPAO2prom-non-uORF::GUS*) [66]. These data suggest that uORF indeed acts as a translational repressor of mORF. Moreover, *AtPAO2* is also modulated in a PA-dependent manner by the uORF located in the 5′UTR [67]. When the PA concentration is low, the *AtPAO2* uORF inhibits mORF translation. Conversely, high levels of PAs increase the expression of *AtPAO2*. It seems that the regulation of PAO gene transcripts by uORFs might be a common mechanism. Recently, a comparative study that explored *AtPAO* orthologous in 24 plant genomes indicated that *PAO* transcripts have one or more uORFs. These findings demonstrate that the translational regulation of PA-related genes mediated by uORFs might be conserved in plants.

Phosphocholine (PCho), one of the main components in cell membranes [68], is not only the precursor of phosphatidylcholine (PtdCho), but also an important metabolite involved in plant development [69]. PCho is synthesized by the catalysis of *S*-adenosyl-l-methionine:phosphoethanolamine *N*-methyltransferase (PEAMT) [68,70]. In *Arabidopsis*, three genes, including *AtXIPTOL1*, encode putative PEAMTs [68,71]. In the *AtXIPOTL1* T-DNA mutant, abnormal phenotypes such as short primary roots, multilateral roots, and short epidermal cells appear, because the biosynthesis of PCho is blocked [72]. A conserved uORF (CPuORF30) has been identified in the 5′-UTR of *AtXIPOTL1* mRNA, which results in the translational repression of mORF just when PCho is at a physiological concentration, without significantly altering its mRNA levels [73]. This hints that a similar mechanism exists in both PCho and PA biosynthesis.

**Figure 2:**
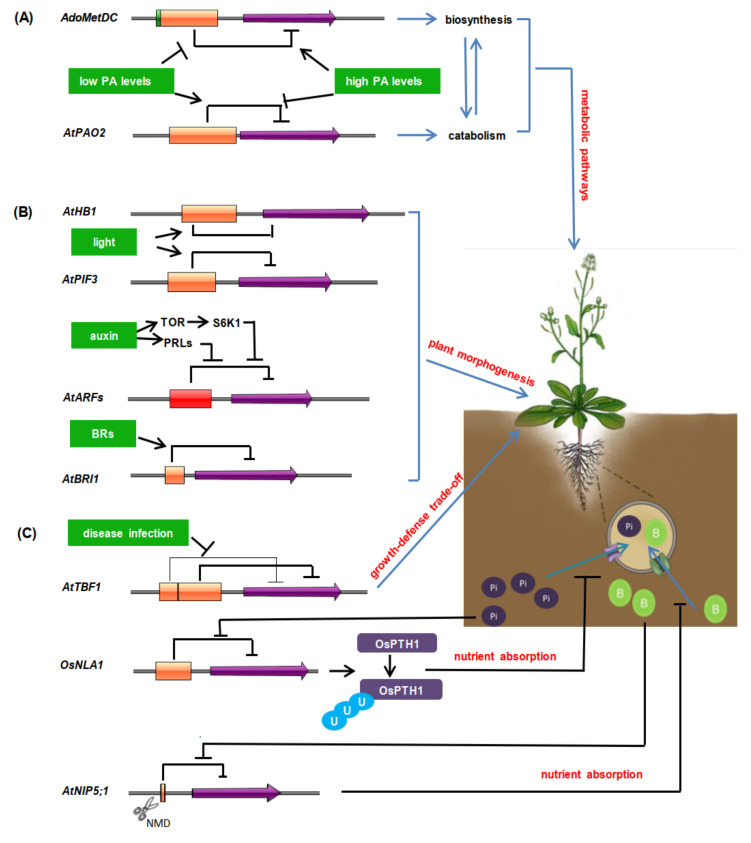
Overview of the regulatory functions of uORFs in plants. (**A**). uORF-mediated translational regulation of plant metabolic pathways [64,66,67]. (**B**). The regulatory roles of uORFs in plant morphogenesis [58,84,87,88]. (**C**). The regulatory roles of uORFs in disease resistance and nutrient absorption [43,89,92]. NMD means nonsense-mediated mRNA decay. The blue circles with “U”, the green circles with “B”, and the purple circles with “Pi” mean ubiquitination, boron, and phosphorus, respectively. Orange boxes represent uORFs, and the box length is proportional to uORF length. Green box in *AdoMetDC* represents tiny uORF, and red box in *AtARF*s means mutiple uORFs. Purple arrows represent mORFs. Black bars indicate negative regulation, black solid lines indicate positive regulation.

Besides PAs and PCho, the translational control of sucrose and vitamin C metabolisms is also mediated by uORFs in a metabolite-dependent manner in plants [74,75]. Some studies have shown that the uORFs involved in metabolic pathways are mostly conservative and sequence-dependent [64,66,73,75], and their activities are always regulated by cellular metabolite concentrations for ensuring metabolite homeostasis. Together, these results fully demonstrate that uORFs play important roles in the translational regulation of plant metabolic pathways (Figure 2A).

### 5.2. Regulatory Roles of uORFs in Plant Morphogenesis

Fine spatial and temporal controls of cell proliferation and expansion sustain differential growth that defines organ shapes and sizes in plants. Accumulating evidences show that translational regulation plays an essential role during plant morphogenesis, which presents an opportunity for understanding a previously underappreciated mRNA function in plant morphogenesis (Table 2).

*AtHB1* (*HOMEOBOX 1*), encoding a member of homeodomain-leucine zipper transcription factor subfamily I, is preferentially expressed in hypocotyls and roots and promotes hypocotyl elongation under a short-day regime in *Arabidopsis* [58,76]. A conserved peptide uORF (CPuORF33) has been identified in the 5′-UTR of *AtHB1* mRNA [9], which has been verified to cause ribosome arrest in vitro or in vivo [58,77]. The activity of CPuORF33 displays tissue- and/or growth condition-specificity. Plants transformed with *35S*:*native*-*uORF*:*GUS* display no obvious phenotype difference in darkness when compared with wild-type plants. However, the opposite occurs under the long-day photoperiod. Further studies show that GUS activity is repressed in aerial tissues, except in darkness, which indicates that the CPuORF33 repressive efficiency is triggered in aerial tissues by light (Figure 2B). In the absence of CPuORF33, the excessive expression of *AtHB1* causes aberrant phenotypes such as serrated leaves, compact rosettes, short or nondehiscent anthers, and siliques containing no or fewer seeds [58]. These indicate that CPuORF33 ensures a relatively low level of *AtHB1* expression in aerial parts and avoids adverse phenotypes. More importantly, CPuORF33 exists in a variety of monocot and dicot species. Its function as a translational repressor in maize has also been identified, which is also tissue-specific [58]. These above results suggest that CPuORF33 may play a conservative role in plants.

Light not only serves as an energy source of photosynthesis, but also acts as an important exogenous environmental signal for plant morphogenesis. In *Arabidopsis,* PHYTOCHROME-INTERACTING FACTOR 3 (AtPIF3) is a basic helix-turn-helix transcription factor [78,79]. AtPIF3 highly accumulates in dark-grown seedlings and then rapidly declines upon light exposure to promote seedling photomorphogenesis [80,81]. A series of studies suggest that the phytochrome B (phyB) photoreceptor induces AtPIF3 phosphorylation to lead to its degradation [82,83], and also affects *AtPIF3* alternative splicing [84]. This phyB-dependent alternative splicing results in the retention of an intron containing a uORF in the 5′-UTR of *AtPIF3* mRNA. In turn, this retained uORF inhibits *AtPIF3* translation (Figure 2B).

The accuracy of hormone levels in plants has led to a thorough study of the regulation of transcriptional levels of hormone-related genes. Surprisingly, they are also regulated at the translation level to enable rapid responses. In the *Arabidopsis* genome, 7 out of 23 auxin response factor (ARF) genes have uORF elements [85,86]. Auxin stimulates the transcription of ribosomal proteins, thus accelerating the translation process of uORF-containing *ARF*s [87]. Additionally, auxin also facilitates the translation reinitiation of *ARF*s through the TOR (target of rapamycin)/S6K1 (S6 kinase 1)-eIF3h (target of rapamycin) signaling pathway [85] (Figure 2B). As for Brassinosteroid receptor protein AtBRI1 (Brassinosteroid insensitive 1), its encoding gene has one uORF in its 5′-UTR [88] (Figure 2B). When exogenous brassinazole is applied, the hypocotyl length of the *uorf* mutant is significantly longer than that of the wild type, because the protein content is increased in the *uorf* mutant. This indicates that uORF_AtBR__I1_ is helpful to maintain the stability of BR (brassinosteroid) level in vivo.

### 5.3. Regulatory Functions of uORFs in Disease Resistance and Nutrient Absorption

As sessile organisms, plants have evolved complex traits to cope with constant biotic and abiotic stresses and show high adaptations to external environment alterations that are buffered by the regulatory interactions of developmental networks. In most cases, uORFs in the 5′-UTR of mRNAs can trigger a less efficient mORF translation and play key roles between the perceptions of environmental factors and subsequent cellular responses.

AtTBF1 (AtHsfB1/AtHSF4), as a member of the heat shock factor (HSF) family, is a major factor in the transformation of plants from growth to defense. It is reported that two uORFs capable of inhibiting the translation of mORF have been found in the 5′-UTR region of its mRNA [89]. Under normal conditions, the translation of *AtTBF1* is inhibited by the two uORFs (Figure 2C). Upon the pathogen attack, the inhibitory functions of uORFs on the *TBF1* translation is released, and AtTBF1 binds to the promoters of defense-related genes to induce their expression [89]. The results suggest that the uORF-mediated translation regulation of *AtTBF1* can rapidly reprogram the gene expression and cope with biotic stress when the plant is exposed to pathogen infection.

Boron is an essential nutrient for plant growth. Low boron leads to a severe loss of crop production, but excess boron is toxic to plants [90]. Therefore, proper boron level is necessary for plant normal growth and environment adaption. *AtNIP5;1* encodes a transporter and is essential for the efficient uptake of boron by roots under low boron conditions. However, under high boron conditions, high expression of *AtNIP5;1* causes excessive absorption of boron [91]. Further studies show that the *AtNIP5;1* transcript has two minimum ORFs (AUG-stops). These minimum uORFs play a crucial role in the boron-dependent regulation of *AtNIP5;1* mRNA. When the plant is boron-deficient, AUG-stops do not function, and the translation of the mORF is reinitiated. By contrast, under high boron conditions, AUG-stops cause ribosome stalling, accompanied by translation suppression of the mORF and mRNA degradation, thus ensuring plants resume normal growth [43] (Figure 2C).

OsNLA1 (NITROGEN LIMITIATION ADAPTATION) has putative ubiquitin E3 ligase activity that mediates the ubiquitination of OsPT1/2/4/7/8/12 in the OsPHT1 (PHOSPHATE TRANSPORTER1) family, which is related with the phosphorus (Pi) absorption and allocation in plants [92]. Alternative splicing at the 5′-UTR of *OsNAL1* generates one uORF-containing transcript, whose promoter activity increases prominently when the Pi supply is above 1.5 mM. It could be seen that, when the Pi concentration is too high in the environment, the *OsNLA1* translation inhibition is removed, promoting the degradation of the corresponding OsPTH1 and avoiding the harms of high Pi in vivo [92] (Figure 2C).

Taken together, these results suggest that uORF-mediated translation regulation develops a way for a rapid alteration of the gene expression during ever-changing environments.

## 6. Prospect of uORFs in Plant Breeding

The aim of plant functional genomics is to first explore the key genes in plant development and to elucidate their regulatory mechanism; then to fine-tune the gene expression and engineer these genes effectively. As a kind of widespread *cis*-element in eukaryotes, uORFs usually suppress the translation of downstream mORFs, which play essential roles in plant development and stress responses. More importantly, most uORFs are tissue-specific and regulated by metabolites or environmental factors [43,58,73]. These regulatory characteristics of uORFs offer a new way for dissection of the gene function and improvement of crop traits.

In plant engineering, the major immune regulatory gene *AtNPR1* (*NONEXPRESSER OF PR GENES 1*) driven by *35S:uORFs_AtTBF1_* is transferred into rice [93], which not only improves the broad-spectrum disease resistance, but also avoids growth retardation caused by overexpressing *AtNPR1*. In this case, the uORF-mediated expression of disease-resistance genes coordinates the tradeoff between plant defense and fitness. In lettuce, CRISPR/Cas9-mediated genome editing of the uORF of *LsGGP2*, a key enzyme-encoding gene in vitamin C biosynthesis, increases the tolerance to oxidation stress and ascorbate content by about 150% [88]. These data indicate the potential value of editing plant uORFs. Even faster, the protocol of editing endogenous uORFs by the CRISPR-Cas9 system has been established and successfully identified in *Arabidopsis thaliana*, lettuce, and tomatoes [88,94]. It is believed that uORFs must have a broader application in the improvement of crop traits in the near future.

## 7. Conclusions

uORFs, short, translated ORFs located in mRNA 5′-UTR, are *cis*-acting elements widely found in eukaryotes that mainly regulate mORF expressions at the translation initiation. Studies have demonstrated the universality of uORFs in plant genomes. Combining bioinformatics and experimental analyses enables us to further identify and to dissect uORF regulatory mechanisms for the genetic improvement of crops.

## Figures and Tables

**Table 1:** The number and percentage of upstream open reading frames (uORFs) in eukaryotes.

Species	Number of uORFs	Number of uORF-Containing mRNAs	Number of Total mRNAs	Percentage of uORF-Containing mRNAs	References
*Arabidopsis*	10,104	5611	15,384	37%	[31]
maize	21,915	7927	26,971	29%	[32]
tomato	1329	1275	20,659	6%	[33]
yeast	10,226	3026	6134	49%	[34]
*Drosophila melanogaster*	35,735	13,135	24,058	55%	[35]
human	17,938	11,670	23,775	49%	[36,37]
mouse	12,450	8253	18,663	44%	[36,37]
zebrafish	-	6053	12,228	50%	[38]

**Table 2:** uORF-containing genes involved in plant morphogenesis.

Gene	Number of uORFs	uORF-Mediated Mechanism	Regulatory Functions	References
*AtHB1*	1	translation initiation	aerial tissue morphogenesis	[58]
*AtPIF3*	1	translation initiation	photomorphogenesis	[84]
*AtARFs*	≥1	translation initiation	auxin signaling pathway, plant morphogenesis	[85,87]
*AtBRI1*	1	translation initiation	BRsignaling pathway, hypocotyl length	[88]
